# Eugenol Inhibits the GABA_A_ Current in Trigeminal Ganglion Neurons

**DOI:** 10.1371/journal.pone.0117316

**Published:** 2015-01-30

**Authors:** Sang Hoon Lee, Jee Youn Moon, Sung Jun Jung, Jin Gu Kang, Seung Pyo Choi, Jun Ho Jang

**Affiliations:** 1 Department of Biomedical Science, Graduate School of Biomedical Science; Engineering, Hanyang University, Seoul, Republic of Korea; 2 Department of Anesthesiology and Pain Medicine, Seoul National University Hospital College of Medicine, Seoul, Republic of Korea; 3 Department of Physiology, Medical School, Hanyang University, Seoul, Republic of Korea; 4 Department of Anaesthesiology and Pain Medicine, Hallim University, Dongtan Sacred Heart Hospital, Kyunggi-do, Republic of Korea; Dalhousie University, CANADA

## Abstract

Eugenol has sedative, antioxidant, anti-inflammatory, and analgesic effects, but also serves as an irritant through the regulation of a different set of ion channels. Activation of gamma aminobutyric acid (GABA) receptors on sensory neurons leads to the stabilization of neuronal excitability but contributes to formalin-induced inflammatory pain. In this study, we examined the effect of eugenol on the GABA-induced current in rat trigeminal ganglia (TG) neurons and in human embryonic kidney (HEK) 293 cells expressing the GABAA receptor α1β2γ2 subtype using the whole-cell patch clamp technique. RT-PCR and Western blot analysis were used to confirm the expression of GABA_A_ receptor γ2 subunit mRNA and protein in the TG and hippocampus. Eugenol decreased the amplitude ratio of the GABA-induced current to 27.5 ± 3.2% (p < 0.05) in TG neurons, which recovered after a 3-min washout. In HEK 293 cells expressing the α1β2γ2 subtype, eugenol inhibited GABA-induced currents in a dose-dependent manner. Application of eugenol also decreased the GABA response in the presence of a G-protein blocker. Eugenol pretreatment with different concentrations of GABA resulted in similar inhibition of the GABA-induced current in a non-competitive manner. In conclusion, eugenol inhibits the GABA-induced current in TG neurons and HEK 293 cells expressing the GABA_A_ receptor in a reversible, dose-dependent, and non-competitive manner, but not via the G-protein pathway. We suggest that the GABA_A_ receptor could be a molecular target for eugenol in the modulation of nociceptive information.

## Introduction

Eugenol is an aromatic molecule present in various plants and essential oils. Numerous studies have examined the biological activity of eugenol as an antibacterial agent and in the immune, reproductive, cardiovascular, gastric, nervous, and urinary systems [1]. Eugenol has been used extensively in dentistry because of its ability to allay tooth pain [2]; however, the molecular mechanisms of its analgesic action remain largely unexplained. Previous studies have suggested that the inhibition of voltage-gated Na^＋^ and Ca^2＋^ channel currents by eugenol might contribute to its analgesic effects [3–5]. In contrast, activation of transient receptor potential vanilloid receptor 1 (TRPV1) and inhibition of voltage-gated K^＋^ channel currents by eugenol might be involved in its stimulatory effects [6]. As a wide range of ion channels are already known to be modulated by eugenol, it could also have other molecular targets.

Gamma aminobutyric acid (GABA) receptors, which are activated by the inhibitory neurotransmitter GABA, exhibit inhibitory activity at the neuronal synaptic membrane in the central nervous system (CNS). They are classified into three types of receptors: GABA_A_, GABA_B_, and GABA_C_. GABA_A_ and GABA_C_ receptors function as anion-selective channels that are permeable to chloride (Cl^–^) ions, whereas GABA_B_ receptors are G-protein coupled receptors [7]. The GABA_A_ receptor in the CNS is a ligand-gated Cl^–^ permeable anion channel that is activated by GABA and mediates fast inhibitory synaptic transmission [8, 9]. The most common subtype of GABA_A_ receptors is the α1β2γ2 type, which accounts for 43% of all GABA_A_ receptors [10] and 90% of the receptors in trigeminal ganglion (TG) neurons [11]. There are two alternatively spliced variants of the γ2 subunit of the GABA_A_ receptor [12, 13]: a short isoform (γ2S) and a long splice variant (γ2L). The long splice variant (γ2L) has an eight amino acid insert (LLRMFSFK) in the major intracellular loop that contains a phosphorylation site (Ser 343) for protein kinase C (PKC). Phosphorylation of this site can alter channel function by negatively modulating GABA-induced currents [14, 15].

GABA_A_ receptors in primary afferent sensory neurons, including TG and dorsal root ganglion (DRG) neurons, are involved in a variety of physiological responses. Previous studies using binding assays [16], immunocytochemical studies [17], in situ hybridization [18], and electron microscopy [19] have found GABA_A_ receptors or their subunits in dorsal root ganglion (DRG) cell bodies and in the central and peripheral processes of DRG cells. The activation of GABA_A_ receptors on a central axon terminal or at a peripheral site results in depolarization known as primary afferent depolarization (PAD) [20] and peripheral PAD, respectively [19]. Thus, these receptors could be associated with the modulation of spinal or peripheral nociceptive transmission.

Previous reports suggested that eugenol potentiates the GABA response in *Xenopus* oocytes expressing the GABA_A_ receptor; this may exert depressant activity on the CNS and strengthen the effect of general anesthesia [21]. To our knowledge, however, there have been no studies investigating whether eugenol directly influences the GABA_A_ receptor in the peripheral sensory system. Therefore, we used the whole-cell patch clamp technique to examine the effect of eugenol on the GABA-induced current in rat TG neurons and in human embryonic kidney (HEK) 293 cells expressing α1β2γ2L and α1β2γ2S.

## Materials and Methods

All experimental procedures for animal use were reviewed and approved by the Institutional Animal Care and Use Committee (IACUC), Medical School, Hanyang University. All procedures were conducted in accordance with the Guide for Care and Use of Laboratory Animals published by the National Institutes of Health.

### Preparation of TG Neurons

TG neurons were prepared from male Sprague-Dawley rats weighing 250 g (Orient BIO, INC., Gapyeong-City, Korea) as previously described [22]. All experimental animals were sacrificed for TG tissue collection. Experimental animals were placed in a plastic chamber (30 cm × 20 cm × 20 cm) and suffocated by isoflurane (Hana pharm, Gyeonggi-do, Korea). Dissected TG were incubated in 3 mL of HBSS (Invitrogen, Carlsbad, CA, USA) containing 0.25% trypsin (Invitrogen) at 37°C for 30 min. TG neurons were dissociated by trituration with a series of sterile Pasteur pipettes, resuspended in TG media containing apyrase (2 U/mL; type VII; Sigma, St. Louis, MO), and plated on glass coverslips that were previously coated with a solution of 0.1 mg/mL poly-L-ornithine (Sigma). TG neurons were maintained in a humidified atmosphere of 95% air/5% CO_2_ at 37°C.

### Cell Culture

HEK 293 cells (CRL-1573; American Type Culture Collection) were cultured in 100-mm culture dishes in Dulbecco modified Eagle’s medium (DMEM; WelGene Inc., Daegu, Korea) supplemented with 10% fetal bovine serum, penicillin G (100 U/mL), and streptomycin sulfate (100 g/mL). For expression of GABA_A_ receptors, HEK 293 cells were transferred to 35-mm culture dishes and transfected with vector pCI using the CaPO_4_ precipitation technique.

### Plasmid Constructs

The plasmid pEGFP-C1 (Clontech, Mountain View, CA) encodes green fluorescent protein (GFP). Rat GABA receptor α1 and β2 expression plasmids were gifts from Dr. Sieghart (Center for Brain Research, Medical University Vienna). These plasmids were constructed in the pCI vector (Promega, Madison, WI) in order to express cDNAs in mammalian cells under control of the CMV promoter. A full-length cDNA clone for expression of human GABA receptor γ2L was purchased from Imagene (Berlin, Germany). A γ2S expression plasmid was generated from the γ2L expression plasmid by deletion mutagenesis using a Quikchange II mutagenesis kit (Stratagene, Santa Clara, CA). Briefly, mutant sequences were generated from the γ2L expression vector by thermal cycling with mutagenic primers using PfuUltra DNA polymerase. The mutagenic primers used for this construction were sense primer 5’-GAA GAA AAA CCC TGC CCC TAC CAT TGA TAT C-3’ and antisense primer 5’-GAT ATC AAT GGT AGG GGC AGG GTT TTT CTT C-3’.

### Receptor Expression

The GABA_A_ receptor cDNAs and GFP cDNA were precipitated for 60 min at 25°C in a 200 μL solution containing 100 mM CaCl_2_, 2 × BES buffered solution (50 mM N,N-bis[2-hydroxyethyl]-2-aminoethanesulfonic acid, 280 mM NaCl, 1.5 mM Na_2_HPO_4_), and 1 g of each cDNA. Co-transfected cDNA mixtures contained either α1β2γ2L and GFP or α1β2γ2S and GFP. The mixtures were added to HEK 293 cells grown on 35-mm dishes and the HEK 293 cells were incubated with the cDNA for 24 hr at 37°C in 5% CO_2_/95% air. After 24 hrs, the cDNA was removed and replaced with fresh culture medium and incubation was continued for 4–72 hrs at 37°C. The cells were transferred to 10-mm glass coverslips 1 hr before performing the electrophysiological recordings.

### Transient transfection of HEK 293 cells

cDNAs encoding the GABA_A_ subunits were expressed in HEK 293 cells cultured in Dulbecco’s modified Eagle’s medium (WelGene, Inc., Daegu-city, Korea) supplemented with 10% heat-inactivated fetal bovine serum containing penicillin and streptomycin (Invitrogen). Cells at 60–80% confluency were used for transient transfection with GABA_A_ cDNAs using the cationic liposome method with 1 μg of supercoiled plasmid cDNA per 1 × 10^5^ cells mixed with 2.5 μL enhancer and 3 μL WelFect-EXTM (WelGene) in 0.5 mL of serum-free medium. After 5 hr at 37°C the medium was replaced with normal HEK 293 medium and the cells were incubated for another 28–36 hr before electrophysiological experiments.

### Electrophysiological recordings

Experiments with TG neurons and HEK 293 cells were performed using the whole-cell patch clamp technique. The patch electrodes and puffer pipettes were pulled from borosilicate capillaries (Chase Scientific Glass, Inc., Rockwood, TN). The resistance of the pipettes was 2–4 MΩ when filled with the solution. Coverslips with transfected cells were transferred to a 0.3-mL recording chamber and perfused continuously with extracellular solution (140 mM NaCl, 5 mM KCl, 2 mM CaCl_2_, 1 mM MgCl_2_, 10 mM glucose, 10 mM HEPES, pH 7.3) at a rate of 30 mL/min. Whole cell patch clamp recordings from fluorescent HEK 293 cells (voltage clamped at -60 mV) were performed at room temperature using an EPC 10 USB amplifier (HEKA Electronik, Lambrecht/Pfalz, Germany). The micropipettes were manufactured from borosilicate glass capillaries (Harvard Apparatus Ltd., Kent, UK) using a PC-10 puller (Narishige Co., Tokyo, Japan), and the resistance of the pipettes was 4–7 MΩ when filled with intracellular solution (140 mM KCl, 10 mM HEPES, 10 mM EGTA, 10 mM glucose, 1 mM CaCl_2_, 2 mM MgCl_2_, pH 7.3).

### Drugs

GABA, eugenol, and GDP-βS were purchased from Sigma (Saint Louis, MO). Eugenol was dissolved in dimethylsulfoxide (DMSO) to make a stock solution, which was diluted to the final concentration in extracellular solution and then applied by gravity through a bath perfusion system. The final concentration of DMSO was less than 0.1% (v/v), which did not affect membrane currents [6]. The perfusion rate of the bath solution was unchanged (4 mL/min) during the experiment.

### RT-PCR and Western blot analysis

RT-PCR

The expression of mRNA encoding GABA_A_ receptor γ2 in the rat TG and hippocampus of 6-week-old rats was confirmed after the removal of non-neuronal components by the addition of araC to the media. Rat TG neurons were prepared in a 350-mm cell culture dish. The medium was exchanged for medium containing 10 μM araC (Sigma) and the cells were maintained for 2 days. The neurons were harvested using a cell lifter for RT-PCR. Total RNA was isolated from the TG and hippocampus using RNAiso Plus (TaKaRa, Japan) according to the manufacturer’s instructions. Total RNA was reverse transcribed for 1 hr at 42.1°C using ImProm-II Reverse Transcriptase (Promega, Madison, WI). After denaturation for 5 min at 94°C, PCR was performed for 30 cycles at 94°C for 30 sec, 54°C for 30 sec, and 72°C for 30 sec.

Western blotting

Samples were mixed with lysis buffer and kept in a cryogenic freezer. Samples were homogenized using a tissue homogenizer and total protein was extracted, separated by electrophoresis, and transferred to a membrane. The membrane was blocked with 5% skim milk for 1 hr, and then incubated overnight at 4°C with rabbit polyclonal antibody against GABA_A_ receptor γ2 (1:250, Abcam, Cambridge, UK) and secondary antibody against rabbit (1:2000, Santacruz Biotechnology, Santacruz, USA) for 1h at room temperature.

### Data analysis

For each drug exposure, whole cell peak currents were analyzed using Pulse program *ver*. 8.67 (HEKA Electronik, Lambrecht/Pfalz, Germany). Dose-response analysis was performed with Origin 6.1 software (MicroCal, Northampton, MA). The normalized GABA-induced current was plotted against the drug concentration and fitted using a standard logistic equation, *E* = *E*_*max*_[C^n^/(C^n^ + IC_50_^n^)], where *E* is the measured effect of the drug, *E*_*max*_ is the maximum effect current, C is the drug concentration, IC_50_ is the drug concentration giving 50% of maximum effect, and n is the Hill coefficient of sigmoidicity. Data were expressed as mean ± SEM and normalized to the control value. Statistical significance was tested using Student’s t-test for paired and non-paired data. A p value < 0.05 (one-tailed) was considered statistically significant.

## Results

### Eugenol inhibits GABA-induced currents in rat TG neurons

**Fig 1:**
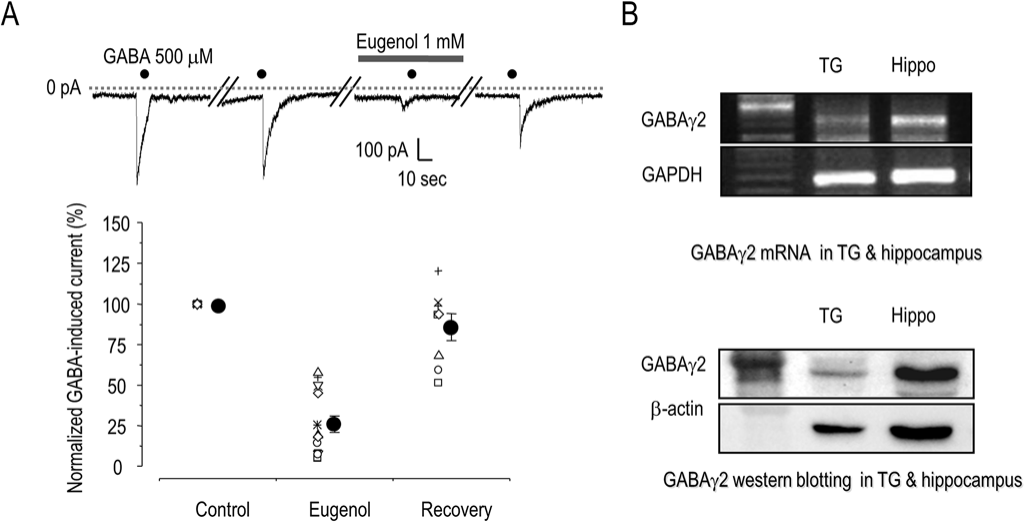
The effect of eugenol on GABA-induced currents in trigeminal ganglion neurons. (A) Eugenol (1 mM) abolished the GABA (500 μM)-induced current in TG neurons; the current recovered to the control value after washout of eugenol (*p < 0.05). (B) The γ2 subunit of the GABA_A_ receptor was expressed in TG neurons. Data are expressed as mean ± SEM.

We first characterized GABA-induced currents using small (< 25 μm) rat TG neurons. After eugenol application (1 mM, 3 min), the amplitude ratio of the GABA (500 μM)-induced current was significantly decreased to 27.5 ± 3.2% of control levels (n = 12, p < 0.05), but recovered to 85.3 ± 3.2% after a 3-min washout of eugenol (n = 8; Fig. 1A). In addition, the current induced by 100 μM GABA was decreased to 34.2 ± 7.0% (n = 9, data not shown) after application of eugenol (1 mM). Next, we checked expression of the α1β2γ2 subtype, which constitutes 50% of the GABA_A_ receptors, in the TG neurons. mRNA encoding GABA_A_ receptor γ2 was detected in the rat TG and hippocampus using RT-PCR. We also observed protein expression of the γ2 subunit in the TG neurons and hippocampus (Fig. 1B), consistent with the previous study [11]. We further confirmed expression of mRNA encoding the GABA_A_ receptor γ2 in the rat TG and hippocampus after removal of non-neuronal components by addition of araC to the media (data not shown). Our results are consistent with a previous study showing that GABA_A_ receptor γ2 subunits are expressed in approximately 90% of TG neurons and that the α1β2γ2 GABA_A_ receptor is the main subtype found in TG neurons [11]. This result indicates that the γ2 subunit of the GABA_A_ receptor in the TG neurons is involved in the GABA-induced current, and that this current was inhibited by eugenol (1 mM) in a reversible manner.

### Eugenol inhibits GABA-induced currents in α1β2γ2 subtype-expressing HEK 293 cells

**Fig 2:**
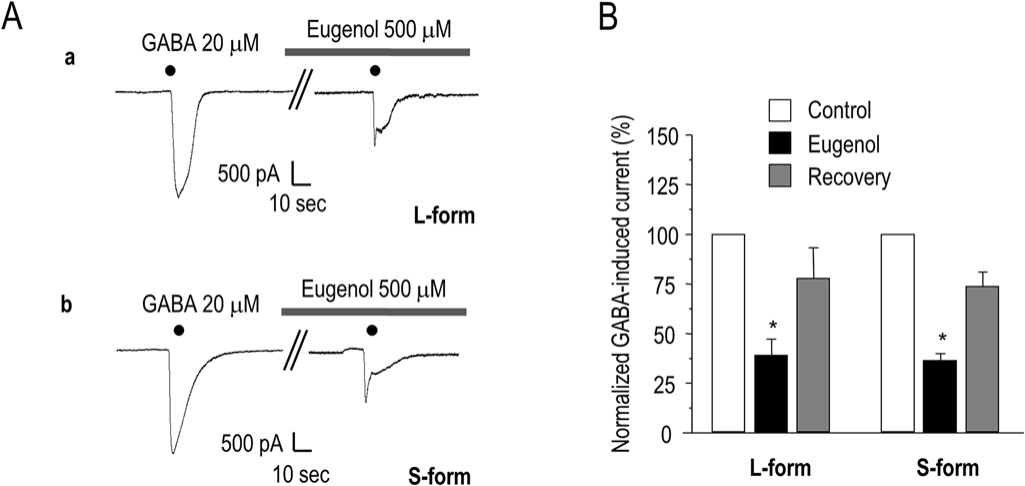
The effect of eugenol on GABA-induced currents in HEK 293 cells with GABA receptors. Eugenol (500 μM) blocked the GABA (20 μM)-induced current. This blocking was observed in both the L- and S-forms of the GABA_A_ receptor (*p < 0.05). Data are expressed as mean ± SEM.

**Fig 3:**
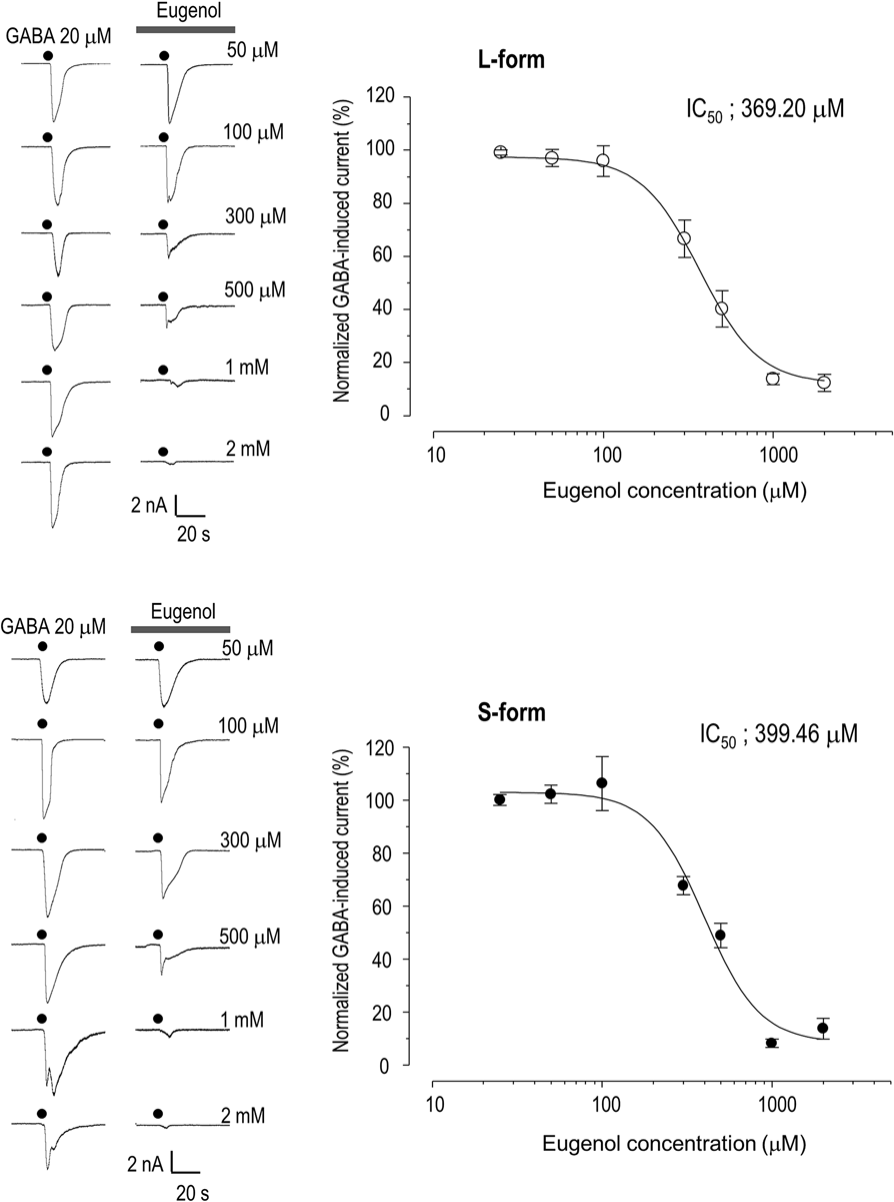
Dose-response curve for eugenol action on GABA-induced currents in HEK 293 cells with GABA receptors. Eugenol (50, 100, 300, 500, 1000, and 2000 μM) blocked the GABA (20 μM)-induced current in a dose-dependent manner. This blocking effect of eugenol on GABA-induced current was observed in HEK 293 cells transfected with the L- or S-form of the GABA_A_ receptor. Data are expressed as mean 00B1 SEM.

We next tested whether eugenol inhibited GABA-induced currents in HEK293 cells expressing the α1β2γ2 subtype. The application of GABA did not induce a current in HEK 293 cells in the absence of GABA_A_ cDNA. In both α1β2γ2L- and α1β2γ2S-expressing HEK 293 cells, however, we observed inward currents induced by GABA (20 μM), which were inhibited by eugenol (500 μM), as in the TG neurons. There were no significant differences between the inhibitory action of eugenol on the α1β2γ2L and α1β2γ2S subtypes (38.1 ± 7.3%, n = 6 and 35.2 ± 3.8%, n = 6, respectively; p > 0.05; Fig. 2). Furthermore, eugenol decreased the GABA-induced current in both subtypes in a dose-dependent manner. The *IC*_*50*_ for inhibition of the GABA-response by eugenol was 369.2 μM for α1β2γ2L and 399.5 M for α1β2γ2S (Fig. 3). This suggests that there is no difference between the GABA responses of α1β2γ2L and α1β2γ2S receptors to eugenol.

### Inhibitory action of eugenol on the GABA response is independent of G-protein

**Fig 4:**
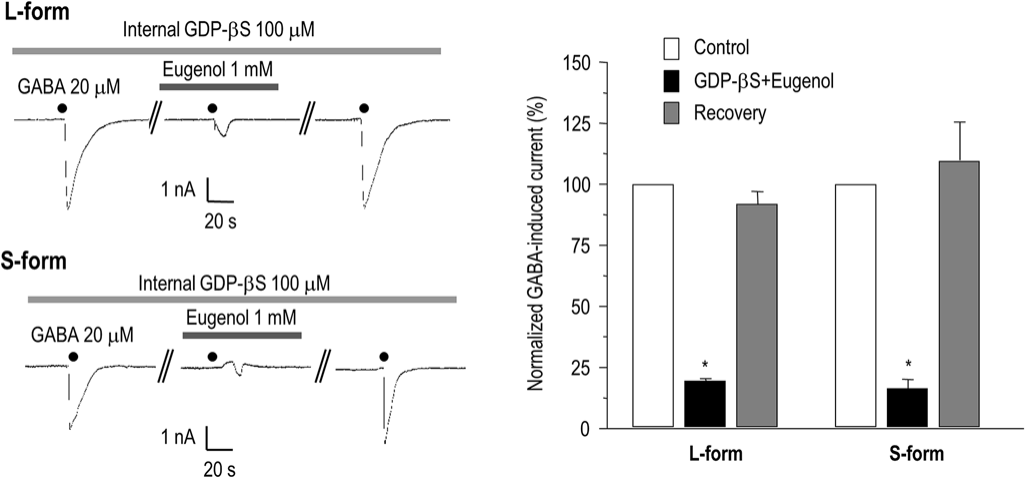
The effect of eugenol on GABA-induced currents in HEK 293 cells expressing GABA receptors was independent of G-protein activation. Eugenol (1 mM) blocked the GABA (20 μM)-induced current in HEK 293 cells transfected with the L- or S-form of the GABA_A_ receptors in the presence of 100 μM GDP-βS (*p < 0.05). Data are expressed as mean ± SEM.

Next, we tested whether the inhibitory effect of eugenol on the GABA response at the α1β2γ2L and α1β2γ2S GABA_A_ receptors was associated with G-protein. We performed whole-cell recordings in cells expressing the α1β2γ2L and α1β2γ2S subtypes with pipette solutions including 100 μM GDP-βS, a G-protein blocker. In the presence of GDP-βS, application of 1 mM eugenol decreased the GABA response to 23.41 ± 1.3% (n = 6) in the α1β2γ2L subtype and 20.2 ± 5.8% (n = 6) in the α1β2γ2S subtype (Fig. 4). This indicates that G-protein is not involved in the inhibitory action of eugenol on the GABA response. In addition, given the similar electrophysiological properties of α1β2γ2L and α1β2γ2S, the effect of eugenol on the GABA response was independent of PKC activation.

### Eugenol blocks the GABA_A_ receptor in a non-competitive manner

**Fig 5:**
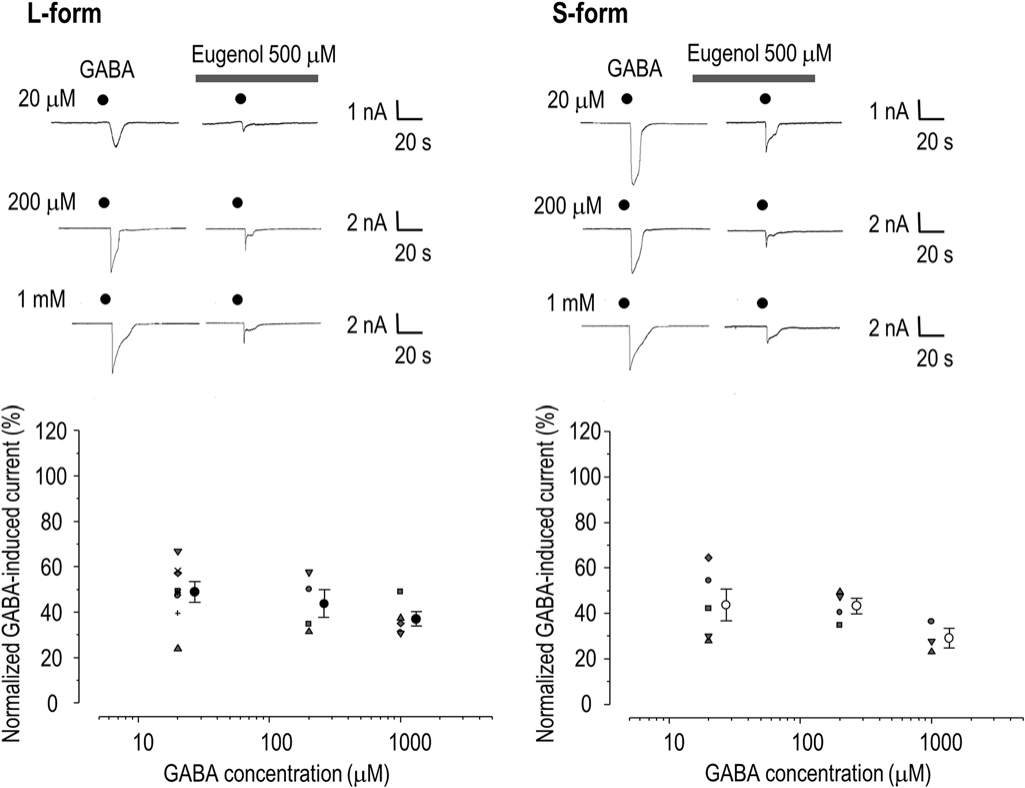
Non-competitive blocking effect of eugenol on GABA-induced currents in HEK 293 cells expressing GABA receptors. Eugenol (500 μM) blocked the GABA (20, 200, 1000 μM)-induced current. This blocking was similar for L- and S-forms of the GABA_A_ receptor (p < 0.05 compared to each GABA concentration). Data are expressed as mean ± SEM.

We further examined the possibility that eugenol directly inhibits GABA_A_ receptors. Pretreatment with eugenol resulted in similar inhibition of the GABA-induced current for different concentrations of GABA (20 μM, 200 μM, and 1 mM; n = 3–8 for each concentration of GABA; p> 0.05 compared to each GABA concentration; Fig. 5). Thus, eugenol significantly inhibited currents elicited by GABA independent of the GABA concentration in HEK 293 cells expressing α1β2γ2L and α1β2γ2S receptors, indicating that eugenol regulates GABA_A_ receptor channel activity in a non-competitive manner.

## Discussion

In this study, we found that eugenol inhibited the GABA-induced current in small TG neurons and HEK 293 cells expressing the α1β2γ2 subtype of GABA_A_ receptor in a reversible, dose-dependent, and non-competitive manner, but not via the G-protein pathway. It has been suggested that eugenol exhibits analgesic activity under some conditions, such as formalin-induced nociception, by blocking the GABA_A_ receptor. This study confirms that the α1β2γ2 subtype of the GABA_A_ receptor could be a molecular target for eugenol, especially in the peripheral nervous system. However, these results are not concordant with those of a previous study, which reported eugenol as a positive GABA_A_ receptor modulator in the CNS [21]. This discrepancy might be caused by differences between the diverse recording systems—*Xenopu*s oocytes, TG neurons and HEK 293 cells. We also observed an inhibitory effect of eugenol on the GABA response using an *ex vivo* recording system in a foot skin-saphenous nerve preparation. The discharges of a single fiber induced by application of 3 mM GABA decreased to 23.1 ± 10.5% (n = 4) of the control value after application of eugenol (data not shown).

GABA_A_ receptors are members of the large ‘Cys-loop’ superfamily of evolutionarily related ligand-gated ion channels [23]. The GABA_A_ receptor in the CNS is responsible for fast inhibitory synaptic transmission [8, 9]. However, activation of the GABA_A_ receptors on primary afferent neurons induces PAD in the central terminal of the axon or on the peripheral site, resulting in inhibition of synaptic transmissions or excitability [19, 20]. Carlton et al. reported that a low dose of muscimol, a GABA_A_ agonist, attenuated the pain response in a formalin test, whereas a high dose of muscimol enhanced the formalin-induced pain behavior. In addition, peripherally applied muscimol resulted in thermal hyperalgesia [19]. Another study showed that activation of peripheral GABA_A_ receptors enhanced nociceptor activity [24]. Recently, GABA_A_ receptors containing the peripheral α_5_ subunit were suggested to play a pronociceptive role in the rat formalin test [25]. These results suggest that GABA_A_ receptors may have a bimodal action in the modulation of peripheral nociceptive transmission.

We found mRNA and protein expression of GABA_A_ receptor γ2 subunits in TG neurons, which play an important role in peripheral pain mechanisms [19]. Our results are consistent with a previous study showing that GABA_A_ receptor γ2 subunits are expressed in approximately 90% of TG neurons and that the α1β2γ2 GABA_A_ receptor is the main subtype found in TG neurons [11]. The GABA-induced current in TG neurons in this study, which was decreased by eugenol, could be mediated by the γ2 subunit of the GABA_A_ receptor. The γ2 subunit of the GABA_A_ receptor has two splice variant forms, a short splice variant (γ2S) and a long splice variant (γ2L). The long splice variant contains a consensus site for phosphorylation by PKC, and phosphorylation of this site negatively modulates GABA_A_ receptor function [15, 26]. Although both splicing variants possess PKC phosphorylation sites at Ser 327, the γ2L subunit has an additional site, Ser 343, at which phosphorylation has a greater effect [26]. However, we observed an inhibitory effect of eugenol on the GABA_A_ receptor in both the α1β2γ2L and α1β2γ2S subtypes, indicating that PKC may not be involved in regulating the effect of eugenol on the GABA_A_ receptor.

Eugenol is commonly used as an analgesic agent in dentistry with few reported side effects, although it can produce adverse local reactions and hypersensitivity. A previous study reported that the analgesic effect of eugenol is associated with the blocking of voltage-dependent Na^+^ and Ca^2+^ channels, the P2X channel, and HCN channel [3–5, 22]. In addition, blockage of voltage-dependent K^+^ channel and activation of TRPV1 have been proposed as underlying mechanisms for the irritative effect of eugenol [6, 27]. With regards to nociception, recent studies have reported nociceptive or anti-nociceptive effects of eugenol. Yano et al. showed an anti-nociceptive effect of methyleugenol on the second phase of formalin-induced pain through the inhibition of NMDA receptor-mediated hyperalgesia via the GABA_A_ receptor [28]. Another study suggested that the anti-nociceptive effect of eugenol might be mediated by the ɑ-adrenergic and opioidergic receptors [29]. However, Abbasipour et al. showed that eugenol itself can induce a nociceptive response, even though eugenol inhibited the induction of nociception by formalin [30]. Taken together, these studies suggest that nociception modulation by eugenol might involve a variety of underlying mechanisms. Because eugenol itself has various bioactive effects, such as the activation of Na^+^, Ca^2+^, and TRPV1 channels, and anti-inflammatory action, it might be difficult to determine the mode of action of eugenol in pain modulation. In this study, direct action of eugenol on the GABA_A_ receptor was observed in TG neurons and HEK 293 cells, which differed from the central action of eugenol on the GABA_A_ receptor that was previously reported. However, it is uncertain whether the activity of eugenol that inhibits the GABA_A_ receptor in the peripheral nervous system results in pain behavior *in vivo*. Further studies are needed to examine the inhibitory activity of eugenol on the GABA_A_ receptor.

There are two possible underlying mechanisms for the inhibitory action of eugenol on the GABA_A_ receptor: (1) eugenol directly blocks the GABA_A_ receptor, or (2) eugenol modulates the GABA_A_ receptor via a cell signaling pathway. Because eugenol can activate a mouse olfactory receptor associated with a G-protein [31, 32], we tested the involvement of G-protein in the eugenol effect. In the presence of GDP-βS, the GABA-induced response was not altered by eugenol, suggesting that the inhibitory effect of eugenol on the GABA_A_ receptor is due to a direct action and not through cellular signaling. Furthermore, eugenol decreased the amplitude of the GABA-induced currents to a similar degree irrespective of the concentration of GABA, which suggests that blocking of the GABA_A_ receptor by eugenol is non-competitive.

In conclusion, our results show that the α1β2γ2 GABA_A_ receptor is expressed in rat TG neurons, and suggest that eugenol might exert inhibitory effects on the α1β2γ2 GABA_A_ receptor. Eugenol inhibited GABA-induced currents in TG neurons in a reversible, concentration-dependent, and non-competitive manner. These results indicate that eugenol might be a novel regulator of the GABA_A_ receptor. As GABA_A_ receptors are primarily involved in the modulation of various physiological and pathophysiological activities in the CNS, the inhibitory effects of eugenol could provide a molecular basis for its pharmacological actions in the nervous system, especially in peripheral pain modulation.
